# XR and mental wellbeing: state of the art and future research directions for the Metaverse

**DOI:** 10.3389/fpsyg.2024.1360260

**Published:** 2024-03-08

**Authors:** Alexandra Taylor, M. Claudia tom Dieck, Timothy Jung, Justin Cho, Ohbyung Kwon

**Affiliations:** ^1^AR and VR Hub, Manchester Metropolitan University, Manchester, United Kingdom; ^2^School of Management, Kyung Hee University, Seoul, Republic of Korea

**Keywords:** XR, metaverse, mental health, wellbeing, bibliometric, SLR, positive psychology

## Abstract

**Introduction:**

The purpose of this study is to provide an overview of extant research regarding XR technology and its effect on consumer wellbeing. With the hopes of informing marketing practitioners on XR consumer psychology, in preparation for the Metaverse.

**Methods:**

To achieve the above aim, two types of analysis took place. Firstly, a bibliometric analysis was conducted which was then followed by a framework-based structured literature review. The latter entailed an analysis of 81 articles evaluated from a positive psychological approach.

**Findings:**

Following the TCCM framework, the analysis revealed the most common psychological theories demonstrating potential avenues for XR to impact consumer wellbeing. Moreover, researchers found preliminary links between, theory, characteristics, and contexts. Giving a preliminary description of how theory manifests into reality. Finally, the overview of extant literature was used to propose new avenues for future research pertaining to marketing, the Metaverse, and consumer effects.

**Conclusion:**

In conclusion, the paper provides stakeholder insights which can ensure minimal consumer risk and sustainable use of the XR technology and Metaverse. While addressing the need for more research that uncovers the psychological effects of emerging technologies, so to prepare for the Metaverse. This is especially important when considering the current upsurge of these technologies and the uncertainties associated with their novelty and the idea of an ‘always on’ consumer.

## 1 Introduction

The introduction of increasingly accessible, immersive technology has brought with it a paradigm shift from traditional passive consumption to rich, immersive interactions (Montagud et al., 2020). This encompasses the availability of extended realities (XR) such as virtual reality (VR) and augmented reality (AR).

Previously, XR research has extended to areas of healthcare (Shaikh et al., 2022; Zhang et al., 2023), education (Zweifach and Triola, 2019), manufacturing (de Giorgio et al., 2023), cultural heritage (Jung et al., 2023a, b) and business (Cranmer et al., 2021). More recently, XR technologies have been implemented within the Metaverse, which refers to a collection of completely immersive digital environments in which users may speak with each other via 3D avatars (Al-Ghaili et al., 2022; Mystakidis, 2022; Xi et al., 2023). Mystakidis (2022) defines this as a post-reality multi-user environment which integrates physical and digital realities; using VR and AR technology to create a multi-sensory, interactive platform. Previously, research has demonstrated XR’s application in treating mental health and its subsequent effect on clinical behavior (Cipresso et al., 2018; Pons et al., 2022). However, there has been minimal discussion of XR’s effect on mental wellbeing. This paucity is furthered due to the novelty of the Metaverse and a lack of academic exploration, emphasizing a need for a holistic investigation of XR in preparation for the quotidian adoption of the Metaverse. Using notions of positive psychology (PP) (scientific study of wellbeing), we can work toward having a more holistic view on XR by exploring its relationship to consumer wellbeing and psychology.

Due to the number of domains XR research transcends, conclusive remarks offer a dispersed view on consumer impacts. Therefore, necessitating a coherent overview of the domain regarding psychology and wellbeing. The current paper aims to cultivate a holistic view by bridging the gap highlighted by an absence of wellbeing and PP in extant considerations of XR. Future derivatives of this study ought to facilitate a seamless adoption of the Metaverse by ensuring stakeholders understand its associated risks and benefits. This paper will contribute to XR and marketing research by providing an overview of current knowledge specific to the domain, theory and method (Kraus et al., 2022). Furthermore, evaluations of literature will highlight unexplored areas for future development of theory, and the conceptualization of new narratives which aim to comprehend the psychological mechanisms underlying XR use. Additionally, we will propose potential pathways for advancing knowledge concerning Metaverse development.

The paper aims to answer the following set of comprehensive research questions:

RQ1 – What are the publication and citation trends within research concerning XR and wellbeing?

RQ2 – What are the most influential publications, sources and authors within XR research from a wellbeing perspective?

RQ3 – What are the major theories underpinning the effect of XR on mental wellbeing?

RQ4 – What are the foremost contexts concerning mental wellbeing within XR research?

RQ5 – What are the major antecedents, mediators and outcomes within research investigating the effect of XR on wellbeing?

RQ6 – What methodologies have been used within research investigating the effect of XR on wellbeing?

### 1.1 Background and conceptualization of XR and the Metaverse

The Reality-Virtuality Continuum proposed by Milgram and Kishino (1994) is generally used as a starting point to classify different immersive technologies. Here, virtual environments (such as Second Life) are non-immersive computer-generated environments in which users can interact with their surroundings and other users – usually accessed via a 2D screen (Penfold, 2009). Similar to virtual environments, VR is defined as an artificial, virtual, viewer-centered immersive experience encapsulated within 3D computer-generated spaces in which a user can navigate and interact – mostly requiring the use of a fully immersive head-mounted display (Flavian et al., 2019; Rauschnabel et al., 2022). Enhanced sensory experiences within VR create a sense of immersion and presence within the virtual environment (Flavian et al., 2019). The prevalence of AR – although less extensive – has more recently been integrated across domains (Cipresso et al., 2018; Jung et al., 2021; Ahmad et al., 2023; Jayawardena et al., 2023). Dissimilar to VR, AR is a hybrid experience that entails an overlay of context-specific virtual content onto the physical environment (Rauschnabel et al., 2022). In between AR and virtual environments on the continuum exists augmented virtuality, which is the addition of an overlay of real-world elements onto virtual environments (Regenbrecht et al., 2004). Augmented virtuality, however, is rarely used or mentioned in the literature (Flavian et al., 2019). Both technologies are similar in their ability to provide interactive, immersive experiences and a sense of presence (Cipresso et al., 2018).

Since the proposal of the Reality-Virtuality Continuum in 1994, technology has advanced greatly and, although it remains a leading taxonomy in the immersive technology field, the continuum now presents various limitations. As Rauschnabel et al. (2022) rightly state, the continuum does not encompass newer terms such as XR and is limited to the use of technical criteria to differentiate between technologies. Considering this, Flavian et al. (2019) present a novel perspective concerning the categorization of immersive technologies and their future counterparts. In their proposal of the ‘EPI’ Cube, three main criteria are used to define and distinguish various immersive technologies: Embodiment (technological), Presence (human), and Interactivity (behavioral).

In light of the recent hype and the struggles with defining the Metaverse, it is useful to compare emerging definitions of the metaverse with the criteria mentioned in the EPI Cub. The Metaverse Roadmap (2017) proposes 4 components of the Metaverse: AR, Life Logging, Mirror Worlds, and Virtual Worlds (Smart et al., 2007). AR refers to the layering of interactive digital information into the real world. Lifelogging refers to the recording of the actions of the users within the Metaverse, and mirror worlds refer to the use of external data to provide an information-rich virtual mirror of the real world. Virtual worlds in this sense refer to the economic and social experiences within the Metaverse and the development of virtual identities. These components are spread across 4 planes: Augmentation, Simulation, Intimate, and External. Augmentation refers to technologies that add new layers or capabilities to the real environment, whereas simulation refers to technologies that create completely new virtual environments that mimic the real world. Intimate refers to technologies that focus on the inward individual or identity, whereas external refers to technologies that focus on the environment in which the user exists.

With this in mind, we can see that the Metaverse is likely to encompass multiple vertices and axes on the EPI Cube, with maximal technological embodiment but varying perceptual presence and behavioral interactivity. The Metaverse is a phenomenon that greatly enhances technological embodiment by both enhancing the real world with digital information as well as providing immersive virtual worlds. Perceptual presence, however, may vary seeing as the Metaverse can involve interactions or experiences within the real world as well as in the virtual world (augmentation/simulation). Furthermore, levels of behavioral interactivity may also vary within the Metaverse, as shown by the intimate/external divide. This paper, therefore, argues that the investigation of the impact of XR technologies such as AR and VR on consumer wellbeing can indeed be extended to reflect the potential realities that the Metaverse may bring about.

## 2 Methodology

As recommended by Paul et al. (2021), the current study conducts both a bibliometric analysis and a framework-based systematic literature review (SLR). Using Biblioshiny (Aria and Cuccurullo, 2017), bibliometric analyses identified hidden patterns in data, conducive to establishing domain foundations and knowledge gaps (Kraus et al., 2022). The SLR review adhered to the TCCM framework (Theory, Characteristics, Context and Method), in a methodological manner utilizing the PRISMA protocol. This allowed for empirical and theoretical observations that were transparent and replicable (Kraus et al., 2022). Furthermore, Paul et al. (2021) argue that framework-based reviews are highly impactful due to increased levels of clarity and coverage. The SLR allowed for the exploration of antecedents, mediators, and outcomes of XR on consumer wellbeing – thus, moving beyond extant literature.

### 2.1 Bibliometric analysis (04/10/22)

Firstly, a performance analysis was conducted which provided descriptive measures of the citation and publication trends of the past decade. Further analyses present the top authors, documents, and sources in terms of relevance and impact. Finally, a co-citation analysis was performed - a science mapping technique that reveals thematic clusters, indicated by co-cited articles. Meeting bibliometric standards, a 10-year publishing radius was included. The combination of performance analyses and science mapping techniques offers readers a complete overview of knowledge that has previously been lacking in marketing and business research (Donthu et al., 2021).

### 2.2 SLR process

#### 2.2.1 Search protocol

To enhance the scientific quality of the SLR, searches were conducted within Web of Science (WOS) and Scopus databases (see Appendix 3), on the 2nd of September 2022 (Loureiro et al., 2021). The original search string consisted of ‘Metaverse’ and ‘Wellbeing’. Unsuitably, this gathered a small total of 24 documents – evidencing inadequate academic exploration of the Metaverse. Instead, the decision was made to fragment the Metaverse into its core technological components of XR technology. The following search string was used accordingly:

‘(Wellbeing) AND (Virtual Reality) OR (Augmented Reality)’.

#### 2.2.2 Inclusion/exclusion criteria

To assist the practicality of conducting the review and ensure relevance, an exclusion criterion was adhered to Appendix 4. As such, publishing dates were limited between 2013 and 2022 to ensure a contemporary review that was in accordance with a more recent trend to research AR (Kraus et al., 2022). An assortment of journal articles and review papers published in the English language were included to limit researcher bias and guarantee a holistic representation of the topic (Aromataris et al., 2015). The inclusion of full texts was decided by relevance to previous wellbeing theories, e.g., Seligman’s PERMA model (2012) and Flow theory (Csikszentmihayli, 1975).

Following the PRISMA method (Appendix 5), duplicates (*n* = 68) were removed using Microsoft Excel, thereafter papers were screened via titles and/or keywords. This provided an opportunity to dismiss papers not relevant to the research objectives.

Similarly, a second screening of abstracts further allowed for the exclusion of irrelevant papers – i.e. reports on physical health. As such, 510 articles were included in the final screening of full texts. During which, the appropriateness of papers was determined by their study aim, design and synthesis, in addition to their accessibility. Elimination of full texts included theoretical feasibility studies, and model/ intervention studies that failed to discuss effectiveness. One study was additionally cut due to design issues and another researching the effect of natural stimuli rather than the incorporation of XR. A total of 81 articles were analyzed.

## 3 Analysis

### 3.1 Bibliometric analysis

#### 3.1.1 Annual growth within the past 10 years (RQ 1)

Since 2013, published XR research has increased, drawing to a peak in 2021 where 29.17% of papers were published. At the time of analysis, 10.2% of papers had been published in 2022. But as the year was ongoing and we have moved into 2023. We expect this percentage to have significantly increased due to ongoing refinement of the Metaverse and XR technology. Moreover, the annual growth rate was measured at 18.28%. Further indicating the expansion of XR research from a psychological perspective.

#### 3.1.2 Overview of published documents (RQ 1)

The analysis included 3,750 documents originating across 1,440 sources (see Appendix 1). The preponderance of journal articles overshadowed the 4.77% of reviews. The average number of citations per year was as follows: 2013 = 3.6, 2014 = 5.6, 2015 = 4.3. This decrease was followed by a steady increase until 2018 (7.5) which has since decreased; 2019 = 7.1, 2020 = 6.8, 2021 = 5.7. This decrease suggests a change in research direction which does not include the current research domain.

Calculation of the journal’s H-indexes revealed the top five most relevant sources, helping answer RQ 2. The hierarchy of journals was determined by the quantity and quality of their outputs. In the first position is *Computers in Human Behavior* (*H* = 37). This was followed by *Computers in Industry* (*H* = 21), *Journal of Business Research* (*H* = 19), *Journal of Retailing and Consumer Services* (*H* = 19), and *Frontiers in Psychology* (*H* = 18). This indicates that attempts have been made to understand consumer technology-related experiences. Interestingly, however, under Bradford Law, *Frontiers* *in Psychology* attracted the most interest from researchers. This could be a foretelling of an upcoming trend to understand consumer psychology in an XR research domain. However, only 2.84% of sources have delved into the relationship between consumer wellbeing and XR technology.

*H*-indexes were again calculated, alongside total citations, to unveil the top 5 most impactful authors. Meaning impact was determined by the number of times published articles match the number of times they have been cited. According to the h-index, the hierarchy of most impactful authors was as follows. In first place ranked Rauschnabel, P. with a H-index of 12 and a total of 1,247. This was followed by, Kim, J. (*H* = 11) (TC = 382), Lee, B. (H = 11) (TC = 489), Lee, J. (H = 11) (TC = 627), and Botella, C. (H = 10) (TC = 484). During analysis, it became apparent that impact cannot be measured by publication numbers solely. For instance, at the time of analysis, the most published author appeared to be Kim, J. (*n* = 28) despite not having the highest H-index. Nor was the impact determined by co-authorship.

#### 3.1.3 Geography (RQ1)

Multiple countries publication (MCP) indicates international collaboration between countries. Measured by the number of times a paper has one or more authors situated in different countries to one another. Therefore, this acts as a reflection of the level of international collaboration each country possesses. The scientific output of each country is also noted. The USA contributes the most to international collaboration (*n* = 1,380) as well as to scientific output. This was followed by China (*n* = 655), UK (*n* = 615), Italy (*n* = 420), and Australia (*n* = 402). Indicating where relevant XR research is taking place. A total of 15 countries have published just one paper relating to XR and psychology. This includes Armenia and Bahrain, perhaps signifying that XR is lesser discussed here.

#### 3.1.4 Influential publications (RQ 2)

Local citation was used to determine the top 10 publications, giving a more accurate representation of the influence these papers have had (Appendix 2). Hilken et al. (2017) take first place with their explanation of how AR-based services enhance customer value perceptions. Followed by Yim et al. (2017) who demonstrated the role of immersion in mediating the relationship between interactivity and vividness and how this promotes joyful experiences. Understandably, two of the most influential papers are authored by Raushnabel et al., evidencing further the impact of this author. Both Rauschnabel et al. (2017, 2019) discuss AR app use, one from a marketing perspective and the other explores the adoption of Pokémon Go. Similarly, another article studied consumer-brand relationships and marketing (Scholz and Duffy, 2018), illustrating how psychology is important in understanding consumer relationships from a marketing perspective.

Four documents researched consumer behaviors and experiences (Javornik, 2016; Poushneh, 2017; Scholz and Duffy, 2018; Flavian et al., 2019), and one article discussed AR in relation to health sciences and anatomy (Moro et al., 2017).

#### 3.1.5 Co-citation analysis and subsequent clustering of themes (RQ1)

**Figure 1:**
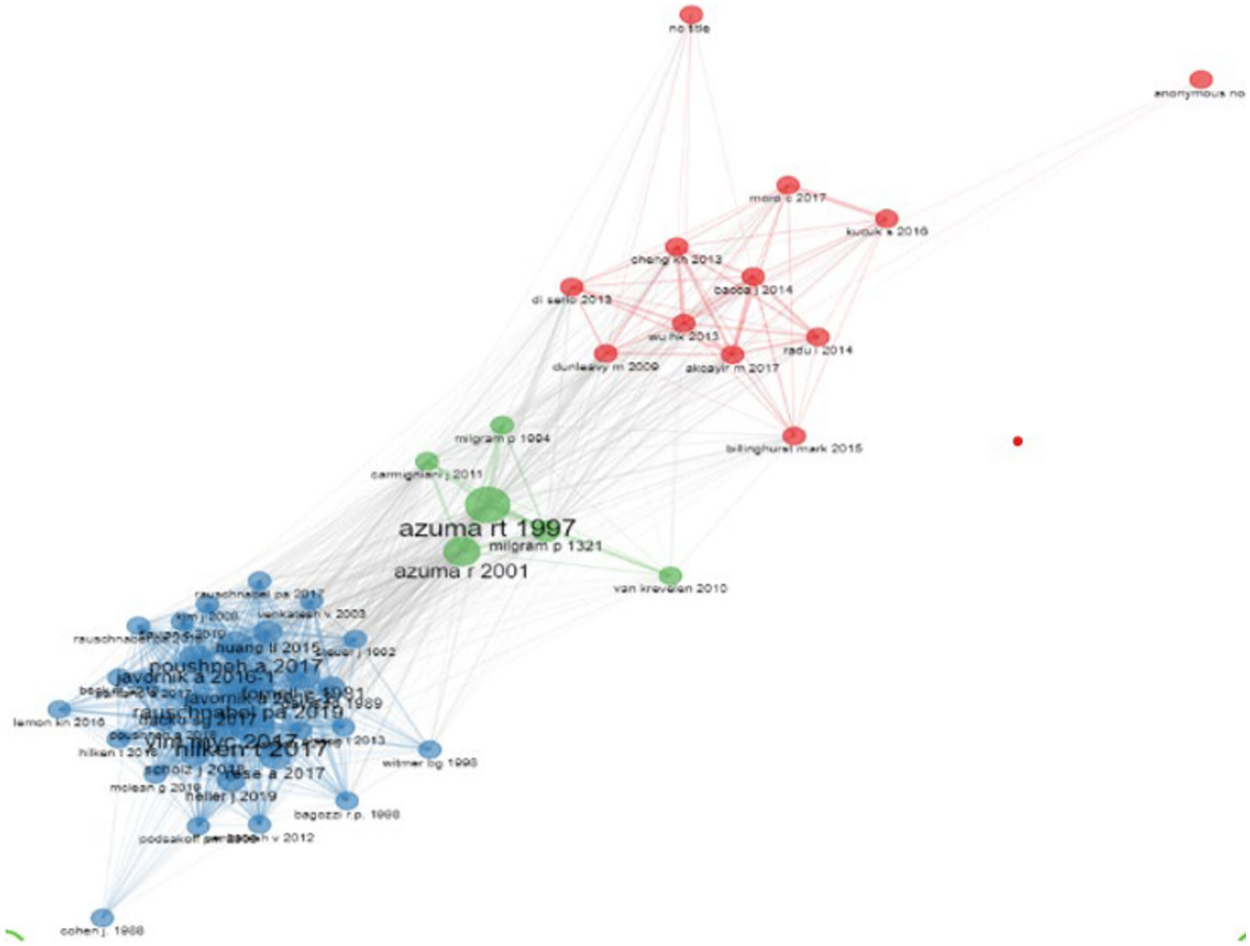
Co-citation analysis.

As exhibited in Figure 1, co-citation analysis revealed three clusters of research. We used the most influential articles of each node to determine the thematic nature of each cluster. The following concepts were identified: applications and limitations of AR with mention of mixed reality and VR, an assessment of AR applied in education and the effect of mixed reality on consumer behavior through a cognitive perspective. The main papers contextualizing the latter cluster are reflective of the top 10 publications (Appendix 2).

### 3.2 TCCM analysis

#### 3.2.1 Theory (RQ3)

The theory was identified using explicit and implicit inferences derived from characteristics and discussions (Kahiya, 2018). We provide an overview of the top 5 most discussed theories in the current domain (Table 1). A total of 61 theories were tallied spanning across a multitude of domains (Appendix 7), including social psychology, restorative psychology, assistive technology, and behavior and functioning. Thirty-eight papers made no explicit mention of theory and 2 spoke of psychological approaches but not theory.

**Table 1:** The five most common theories found in data.

Theory	Number of articles (implicit + explicit)	Explicit examples	Implicit examples
Hedonic (Kahneman et al., 1999)	23 (10 + 13)	Kitson et al. (2018), Yaden et al. (2018), and Barreda-Angeles and Hartmann (2022)	Appel et al. (2021), Li et al. (2021), and Wingenbach and Zana (2022)
Eudaimonic (Ryan and Deci, 2001)	14 (4 + 10)	Gaggioli et al. (2016), Riva et al. (2020), and Laato et al. (2021)	Javornik (2016), Pomianowska (2018), andWilliams et al. (2021)
Uses and Gratification (U&G) (Katz et al., 1973)	11 (8 + 3)	Javornik (2016), Rauschnabel et al. (2017), and Kaimara et al. (2022)	Kothgassner et al. (2017), Kosa et al. (2020), and Xu et al. (2021)
Stress reduction (SRT) (Ulrich et al., 1991)	11 (4 + 7)	Alyan et al. (2021), Karacan et al. (2021), and Li et al. (2021)	Watanabe et al. (2017), Riches et al. (2021), and Newman et al. (2022)
Flow (Csikszentmihayli, 1975)	10 (5 + 5)	Rauschnabel et al. (2017), Kitson et al. (2018), and Ku et al. (2021)	Wang et al. (2019), Siani and Marley (2021), and Torous et al. (2021)
Other theories	59	Riva et al. (2016), Orosz et al. (2018), and Miller et al. (2021).	

##### 3.2.1.1 Hedonism

Predominately, references to hedonism were made throughout XR literature – hedonism theorizes that happiness is attained through an increase in positive emotion and life satisfaction met by reduced negativity. XR research specifically utilized this to exemplify the potential of immersive technology to assist in hedonic wellbeing. Specifically, social VR encourages interpersonal satisfaction through its ability to promote gratitude (Collange and Guegan, 2020). Barsasella et al. (2021) similarly evidenced the power of XR in combating issues of aging thus enhancing the life satisfaction of the elderly. Showcasing how XR can promote positivity and satisfaction by reducing real-world negative stimuli. It would be interesting to uncover to what extent satisfaction and happiness transcends into users’ real life. Asking the question of whether effects are short-term or long-term. Additionally, as research has shown XR’s ability to induce positivity, it is worth future research to assess if it can also induce negative affective states. Therefore, promoting positive psychology through the identification and management of risk.

##### 3.2.1.2 Eudaimonism

Although less explicitly mentioned, XR research often hinted toward concepts influencing eudaimonic happiness. Equivocal discussions of eudaimonism use environmental mastery, achievement, meaningful experiences, and self-realization as antecedents of positive personal growth. AR games in particular provide consumers with a sense of achievement which fosters a sense of self-fulfillment (e.g., Ku et al., 2021). Furthermore, XR activities can supply meaningful experiences by promoting social wellbeing and imagination (Laato et al., 2021). Not only does this show how XR can address eudaimonic needs, but also indicates the satisfaction of needs in general. Moreover, 11 papers discussed hedonic and eudaimonic traits of happiness simultaneously. This suggests a link between the two philosophies and that positive self-transcendent emotions increase the meaningfulness of XR activities (e.g., Gaggioli et al., 2016). However, what happens when eudaimonia is met by negative hedonic states? The nuances of the relationship between hedonic and eudaimonic should further be explored. Furthermore, Kosa et al. (2020) stated that environmental mastery fosters intrinsic motivation and explained how this can improve wellbeing.

##### 3.2.1.3 Uses and gratification theory

In its general application, U&G illuminates how specific forms of media satisfy specific psychological needs. This was a prevailing theme throughout the current dataset that discusses AR technology (e.g., Rauschnabel et al., 2017; Laor, 2020). Papers revealed explicit links between U&G and AR gaming, immersion, and AR face filters (e.g., Javornik, 2016; Laor, 2020). Primarily these examples suggest further the notion that AR can gratify social needs, as well as supplementary needs of achievement, escapism, and positive self-reflection. In addition to explicating how XR improves wellbeing, research pinpoints gratification of needs as a motivator for the continual use of XR, identifying immersion as a key influence (e.g., Kosa et al., 2020).

##### 3.2.1.4 Stress reduction theory

SRT is used to explain the restorative effect of natural stimuli in reducing psychological stress (Shaffee and Shukor, 2018). Within the current dataset, there was a trend to focus on how XR can facilitate this (e.g., Alyan et al., 2021; Jo et al., 2021). It was common for mentions of SRT to be alongside discussions of attention restoration theory (e.g., Karacan et al., 2021; Newman et al., 2022). Suggesting that in addition to a calming effect, virtual environments can also reduce mental fatigue and concentration (e.g., Seabrook et al., 2020; Jo et al., 2021). By reducing negative emotions such as stress and fatigue, SRT allows for hedonic wellbeing (Li et al., 2021). Although SRT traditionally focuses on natural stimuli, papers have shown how alternative virtual environments can reduce stress (e.g., Torous et al., 2021; Schrempf et al., 2022). Proposing potential new methods of stress reduction that use XR technology to foster hedonic wellbeing. Using this theory, research should assess how beneficial XR will be as a method of stress reduction in a clinical setting. Perhaps this will facilitate in the implementation of XR in clinical settings. This could support attempts made in positive psychology to ease the symptoms of mental health conditions.

##### 3.2.1.5 Flow theory

Flow theory was consistently noted throughout current data, on topics such as mental health, positive technology and mindfulness (e.g., Riva et al., 2020; Seabrook et al., 2020; Alyan et al., 2021). Traditionally, Flow is an increase in engagement which is known to facilitate personal capacity and goal achievement. A past study found improvements in self-efficacy (personal capacity), reduced self-criticism and subsequent negative affect (Kaimal et al., 2020). This demonstrates how engagement with XR can facilitate personal capacity and promote hedonic wellbeing. Again, this highlights the ability of XR to gratify eudaimonic needs. As well as supporting preliminary links between hedonic and eudaimonic wellbeing that should be investigated further. Similar to the discussion of U&G, the data highlighted the immersive quality of XR as a direct constituent of Flow (e.g., Kosa et al., 2020; Jang, 2021). Suggesting immersion to be a causal feature of XR to produce an effect on wellbeing. Subsequently, to direct XR use in a way that ensures positive wellbeing, we must understand how causal mechanisms, such as immersion, mediate the relationship between technology use and consumer wellbeing.

#### 3.2.2 Context (RQ4)

We considered the major wellbeing contexts of XR research in relation to previous inter- and intra-personal levels of wellbeing. These are all presented in Table 2.

**Table 2:** Wellbeing contexts.

Context	*N*	Citation example
Emotional wellbeing	21	Newman et al. (2022)
Psychological wellbeing	14	Laor, 2020
Social wellbeing	4	Epting (2021)
Cognitive wellbeing	3	Gao et al. (2022)
Social and psychological wellbeing	8	Lee et al. (2021)
Emotional and psychological wellbeing	7	Li et al. (2021)
Emotional and social wellbeing	6	Collange and Guegan (2020)
Cognitive and emotional wellbeing	4	Pomianowska (2018)
Cognitive and psychological wellbeing	1	Rauschnabel et al. (2017)
Spiritual wellbeing	9	Alyan et al. (2021)
Physical wellbeing	3	Miller et al. (2014)
Cognitive and physical wellbeing	1	Watanabe et al. (2017)

Similar to Chakma et al. (2021)‘s use of the TCCM framework, the study shows how contexts can interlink and be apparent together as well as alone.

##### 3.2.2.1 Emotional wellbeing

Evidently, research assesses consumer experience in relation to emotional wellbeing– focusing on how XR can be used to induce emotion and reduce stress. This was clear in intervention studies that operated alongside therapy and a restorative nature. Furthermore, the literature depicts a social element in emotional wellbeing – represented by the overlap of contexts presented in Table 2. Similarly, there also appears to be an overlap between emotional wellbeing and physical activity.

##### 3.2.2.2 Psychological wellbeing

Alternatively, contexts of psychological wellbeing were depicted by stipulations of eudaimonic and hedonic happiness. This implies that wellbeing has been measured through eudaimonic constructs such as self-acceptance, personal growth, and environmental mastery. As well as hedonic measures that report positive emotions and life satisfaction. The data reveals that awe, achievement, and engagement are just some of the contextual effects incorporated into psychological wellbeing.

##### 3.2.2.3 Social wellbeing

A portion of the research investigated the effect of XR in the context of social wellbeing; relating to social relationships, acceptance, contribution, and social actualization (Salehi et al., 2017). These contexts emphasized the importance of perceived authenticity and social connectedness in facilitating wellbeing.

##### 3.2.2.4 Cognitive wellbeing

Although less prevalent, there was also a discussion of cognitive wellbeing. Congruent with psychological wellbeing, satisfaction is used as an indicator of wellbeing. However, in a more localized manner – for instance, work-life satisfaction (Luhmann et al., 2021). This again insinuates overlap between contexts that suggest improvements in one context may impact another context. However, more research that is tailored to wellbeing attained in specific life domains should be conducted to establish how contextual use affects each context of wellbeing.

##### 3.2.2.5 Spiritual wellbeing

Ryff (2021) proposes that spirituality and wellbeing are mediated by interactions with nature, art, and literature. Therefore, this indicates that the papers studying restorative nature and art therapy using XR technology are also assessments of spirituality. This can be understood further using eco-existential positive psychology. This depicts the effect of nature on identity, happiness, and social connectedness (Passmore and Howell, 2014). Alongside the evidence that supports SRT, XR should be assessed as a tool in stress reduction to identify its role in clinical settings.

##### 3.2.2.6 Physical wellbeing

Furthermore, the data comprised articles which discussed the effect of physical activity and XR in contributing to mental wellbeing. These included studies of AR gaming golfing and cycling in virtual reality.

These contexts demonstrate a fragmented review of wellbeing whilst providing preliminary evidence of overlaps between contexts. This further provides the notion that researchers lack a consensus when defining contexts of wellbeing (Mansfield et al., 2020). Providing a potential research agenda that investigates XR’s holistic effect on wellbeing. So to develop a standardized extension of wellbeing theory into an XR domain. Unfortunately, the current review fails to depict alternative contexts such as subjective wellbeing – and cannot comment on the generalizability of studies conducted within COVID-19 (*n* = 6), offering further pathways for future consolidations.

#### 3.2.3 Characteristics (RQ 5)

Loureiro et al.’s (2021) analysis revealed that the characteristics formed conceptual constituents of the theories discussed above. A link between characteristics meant that antecedents, mediators, and their outcomes were easily identified.

##### 3.2.3.1 Social connectedness

Social connectedness appeared ubiquitous within the data and seemed an essential element across a range of wellbeing theories. More often, social connectedness was measured as an outcome (Miller et al., 2014; Laor, 2020; Wingenbach and Zana, 2022). As well as a mediating variable (Watanabe et al., 2017; Orosz et al., 2018; Laor, 2020) and an independent variable (Baker et al., 2019; Ewell et al., 2020). Most studies revealed increases in social connection when using XR technology (e.g., Miller et al., 2014; Barreda-Angeles and Hartmann, 2022). This was assumed to be due to increases in shared experiences (Laato et al., 2021; Xu et al., 2021). However, not all research found that social connection was beneficial and instead warned of social isolation and anti-social behaviors (Kaimara et al., 2022).

##### 3.2.3.2 Negative affect and positive affect

Further remarks of negative affect warned of addiction (Hussain et al., 2018; Grajek et al., 2022; Usmani et al., 2022), anti-social behaviors (Kothgassner et al., 2017; Kaimara et al., 2022), and detachment (Kaimal et al., 2020; Karacan et al., 2021). Positive outcomes were attributed to enhanced psychological intervention and reductions in stress, anxiety, and depression (Tanja-Dijkstra et al., 2014; Kogan et al., 2017; Newman et al., 2022). These emotional outcomes were in relation to XR’s ability to enhance mindfulness, meditation, and relaxation (Yaden et al., 2018; Ellis et al., 2020; Crosswell and Yun, 2022). Discussions of physical activity were evenly distributed as an antecedent (Kogan et al., 2017; Wang et al., 2019), mediator (Singh et al., 2017; Ellis et al., 2020), and an outcome (Ku et al., 2021; Siani and Marley, 2021) of XR. Congruent with theory, studies also reported increases in life satisfaction (Laor, 2020; Jang, 2021; Xu et al., 2021).

##### 3.2.3.3 Enhancing interventions

Investigations of psychological intervention delivered through XR recurrently emerged within screening. Predominately, these studies supported the feasibility and effectiveness of VR-mediated therapies (Wrzesien et al., 2015; Kaimal et al., 2020; Jeppesen et al., 2022), alternative methods of intervention (Epting, 2021; Matsumoto et al., 2021; Crosswell and Yun, 2022), and within restorative nature (Schebella et al., 2020; Alyan et al., 2021; Williams et al., 2021). The most common finding of these studies was an increase in accessibility (Barsasella et al., 2021; Jang, 2021; Riches et al., 2021).

##### 3.2.3.4 Presence, immersion, and engagement

Presence, immersion, and engagement additionally appeared habitually throughout the data (Vogt et al., 2015; Dirin and Laine, 2018; Miller et al., 2021), allied with a discussion of Flow and eudaimonism. The data depicts a storyline in which emotion induction is determined by presence and immersion (Mcintosh et al., 2019; Kosa et al., 2020; Schebella et al., 2020). In certain studies, these characteristics motivated the continual use of XR technology (Meneses-Fernandez et al., 2017; Kosa et al., 2020; Laato et al., 2021). Congruent with theory, engagement motivated by escapism was mentioned throughout contexts; AR gaming (Laor, 2020), clinical intervention (Wang et al., 2020) and achievement/self-fulfillment (Orosz et al., 2018; Ellis et al., 2020; Grajek et al., 2022), further evidencing the application of eudaimonism in answering RQ 3 and 4.

##### 3.2.3.5 Socio-economic divide

Despite suggestions of increased accessibility for clinical populations (Singh et al., 2017; Thabrew et al., 2022), certain studies depicted a socio-economic divide within emerging technologies (Meneses-Fernandez et al., 2017; Riches et al., 2021; Usmani et al., 2022). Causing concern over the Metaverse’s ability to add to this divide – it is therefore important for proprietors to acknowledge this if they wish to avoid a negative impact on societal wellbeing.

#### 3.2.4 Methodology (RQ6)

Sample sizes varied from *n* = 4 (Wrzesien et al., 2015) to *n* = 2,530 (Watanabe et al., 2017) and incorporated an array of sample populations. A tendency to use quantitative methods (56.79%, Appendix 6) was found, including the use of randomized control trials (e.g., Barsasella et al., 2021) which measured the effectiveness of XR interventions. Similarly, pre-test/ post-test intervention studies were used to measure changes caused by XR intervention (e.g., Singh et al., 2017). Data collection strategies varied and were inclusive of surveys, questionnaires, and clinical inventories.

Qualitative methods consisted of mainly literature reviews (*n* = 17) (e.g., Gaggioli et al., 2016; Appel et al., 2021). Data collection mainly consisted of interviews (*n* = 4) (Ku et al., 2021), followed by questionnaires (*n* = 2) (Arjoranta et al., 2020) and narrative feedback (*n* = 1) (Kaimal et al., 2020). One study additionally utilized researcher notes and a focus group (Baker et al., 2019). Thematic analyses took precedence during analysis (*n* = 6) (Laor, 2020), with one study conducting a content analysis (Ku et al., 2021).

Mixed methods (*n* = 12) tended to be an amalgamation of statistical and thematic analysis (Ellis et al., 2020), or self-report measures, surveys and/or questionnaires (Meneses-Fernandez et al., 2017; Kaimal et al., 2020). However, these types of studies were lacking in comparison to quantitative and qualitative methods.

This overview highlights a gap for varied research methods to be employed. For instance, Loureiro et al. (2021) explain how content analyses and text mining can evaluate online media and so can offer new perspectives to the domain.

## 4 Discussion

**Figure 2:**
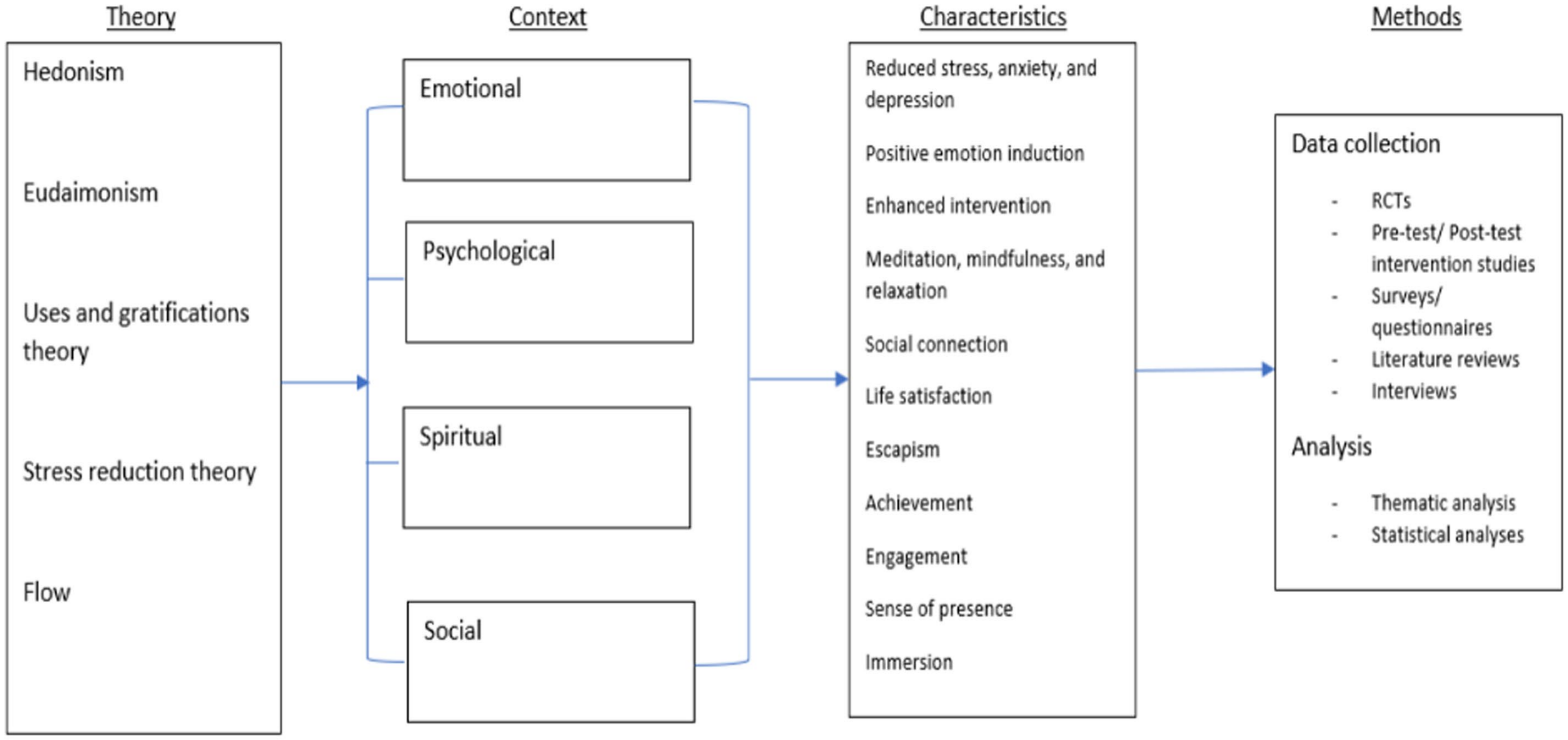
Proposed XR/Metaverse mental wellbeing framework.

The bibliometric analysis revealed a current shift in XR research focus, toward a psychological perspective (RQ1 & 2), as indicated to by Bradford’s Law. Moreover, Adhering to a TCCM framework afforded an overview of extant literature; specific to the psychological fundamentals of wellbeing and how the use of XR can facilitate this. Whilst revealing popular theories, contexts, characteristics, methods and links between each. This informed the framework presented in Figure 2, which aims to enhance stakeholder knowledge regarding wellbeing – in response to an increased adoption of XR within Metaverse development. It is hoped that the information presented can be used to market XR in a way that aligns with positive psychology.

Theory: 61 theories stemming from varied psychological approaches, i.e., social, cognitive, emotional, and behavioral were revealed (Appendix 7). Additional theories related to human functioning, motivation, and engagement. This diversity complicates attempts to develop a cohesive theoretical framework and highlights a disjointed view of the effects of XR on consumer wellbeing.

However, utilizing the top five theories, we can begin to understand how XR technologies, and their role in the Metaverse, can impact consumer wellbeing. Firstly, using SRT, we know that virtual stimuli reflective of natural environments have a calming effect and reduce negative emotions felt by its viewers. Additionally, XR and Metaverse experiences can be tailored to satisfy the unique needs of its users such as, social connection, escape, and a need for achievement. Finally, in keeping with the Flow theory, XR’s ability to enhance engagement positively relates to a person’s perceived personal capacity and goal achievement. The question then becomes, how do these theories relate to a person’s well-being?

The above theories are further reflective of hedonic and eudaimonic wellbeing.

Hedonia assumes XR’s ability to reduce negative emotions (e.g., SRT) and promote life satisfaction through the gratification of needs (e.g., U&G theory) increases the user’s wellbeing. This is due to its assumption that positive experiences and emotions, matched by a general satisfaction with life equates to wellbeing, On the other hand, the consensus between eudaimonic theorists indicates that XR can facilitate wellbeing as it offers users an environment that promotes positive social relationships, achievement, and personal capacity (e.g., Flow). Both these hedonic and eudaimonic constructs are reflective of Seligman’s PERMA model (Seligman, 2012). Therefore, the positive effect of XR and the Metaverse on consumer wellbeing is perhaps due to their ability to foster positive emotion, engagement, relationships, meaningfulness, and accomplishment (See Seligman, 2012). As such, hedonic and eudaimonic measures become an indicator of XR’s, and the Metaverse’s, capacity to foster a positive psychology.

Contexts: Theory was applied throughout varying contexts of wellbeing with emotional wellbeing taking the forefront. The overview works toward providing a holistic understanding of how XR informs wellbeing by considering the many contexts this occurs in Chan and Hazan, 2022. Predominant discussions of emotional and psychological wellbeing are further reflective of hedonism and eudaimonism. Thus, this study demonstrates how theory contextualizes wellbeing.

Characteristics: Furthermore, the TCCM identified the respective outcomes of different antecedents and mediators. These characteristics allowed us to decipher both explicit and implicit uses of theory Moreover, by acknowledging both contexts and characteristics we can make theoretical predictions based on the mediating variables associated with a particular context. In this way, we can begin to understand how the integration of XR within the Metaverse can impact consumer wellbeing. For example, literature which measures enhanced psychological wellbeing often assumes that this is due to increased life satisfaction and/ or achievement, brought about by the immersive experiences and increased engagement that XR offers. As previously discussed, satisfaction and achievement relate to hedonic and eudaimonic philosophy. Therefore, by applying these theories we can determine that the Metaverse and XR affect psychological wellbeing by satisfying hedonic and eudaimonic needs. However, one characteristic is not exclusive to one context. Social connectedness, for example, relates not only to social wellbeing but also to psychological and emotional wellbeing. This reiterates the fact that the Metaverse and XR can affect a range of wellbeing contexts but also highlights its power to do this at one time.

Methodology: There has been a proclivity to engage in quantitative and qualitative methods of research, with a majority for quantitative methods. Quantitative methods, when studying the effect of XR, create objective, numerical results where changes and differences can easily be identified (Basias and Pollalis, 2018). However, there is an argument that quantified texts reduces the quality of derived comprehension and analysis of a phenomenon, and therefore, that qualitative methods may be more appropriate (Basias and Pollalis, 2018). These offer in-depth knowledge which can facilitate new areas of research. Despite this, qualitative methods may be influenced by research subjectivity and so cannot offer objective, numerical results ready for generalization (Basias and Pollalis, 2018). There was some use of mixed methodologies, which may overcome the downfalls of mono-quantitative and qualitative methodologies by integrating their advantages. The array of contexts relating to wellbeing, sample size and demographic populations is an acclamation to the review’s external validity (Avellar et al., 2016). This is often considered less commonly within SLRs in comparison to internal validity (Avellar et al., 2016).

### 4.1 Limitations and future directions

The bibliometric analysis revealed an opportunity for imminent research to facilitate a change in research direction. This suggests that future research should attempt to close the gap in knowledge by adopting a psychological perspective when further evaluating the effect of XR, and the Metaverse, on consumer wellbeing. The combination of performance analyses and science mapping techniques within the current bibliometric analysis, offers a complete representation of knowledge in comparison to previous business research (Donthu et al., 2021); which can be used to inform and justify new directions in future research including those that take place in a marketing space. However, readers should be mindful that papers require 2–3 years to accumulate enough citations for bibliometric analyses to be reliable (Belter, 2015). Thus, this should be considered when using the bibliometric conclusions of studies published between 2019 and 2022.

Preliminary links between theory, context, and characteristics, suggest that characteristics are the result of theory and context. However, as with most qualitative studies, there is a risk of reporter and selection bias (Drucker et al., 2016), especially in the implicit identification of theory. In attempts to overcome this, the researcher employed the PRISMA method and techniques conducted within high-quality, peer-reviewed papers to enhance internal validity and reduce bias (e.g., Loureiro et al., 2021). However, these findings require replication to ensure their validity.

Future research should not overlook the additional theories presented in Appendix 7, as these may be more applicable as the Metaverse develops. For instance, the Self-determination theory offers a reputable investigation of how motivation to use technology can impact consumer experience and wellbeing (Ryan and Deci, 2001). Furthermore, the current review proposes the adoption of mixed-method studies will be conducive to facilitating research that is both generalizable and detail-rich (Basias and Pollalis, 2018).

Moreover, the tendency for literature to review positive characteristics of XR and the Metaverse ignores lesser studies which depict their detrimental effects. Specifically, these relate to anti-social, self-isolative and addictive behaviors. Thus, future research should consider how the duality of XR and the Metaverse affects their ability to induce both negative and positive wellbeing. This would require an investigation of the underlying properties belonging to XR and the Metaverse, such as presence and immersion, and how these can negatively and positively affect wellbeing.

Lastly, the delineations of this study are wholly theoretical and although they provide a foundation for the study of XR and the Metaverse through a psychological perspective, more research is needed to establish their truth. With this, research should establish the extension of these theories within an XR and Metaverse context. For example, stakeholder perspectives that depict the applicability of these theories within a Metaverse context should be obtained. Moreover, structural equation modeling which enables us to establish the relationship between variables (Hair et al., 2021) could be explored. These will illustrate the specific properties and characteristics of emerging technologies that are responsible for specific wellbeing constructs. More specific research questions are listed below.

### 4.2 Practical implications

Both above and below discuss the theoretical implications of this literature review in relation to how findings can further the otherwise novel research domain. In addition, the findings of this project are of practical use, especially within the XR and Mental health industries. As this paper demonstrates the potential for technology to interact and influence user wellbeing, it provides insights that are to be considered during XR technology and application development. This should motivate industry to take a proactive approach in ensuring user mental health. Moreover, using theory, industry could devise applications that are targeted for specific mental health conditions. Integrating technology into mental health services provides new methods of treatment and recovery, which could potentially be used to bypass current clinical wait times. These are just a couple of ways the insights in this research can be practically applied to industry to ensure positive consumer wellbeing is achieved.

### 4.3 Future research questions

**Figure 3:**
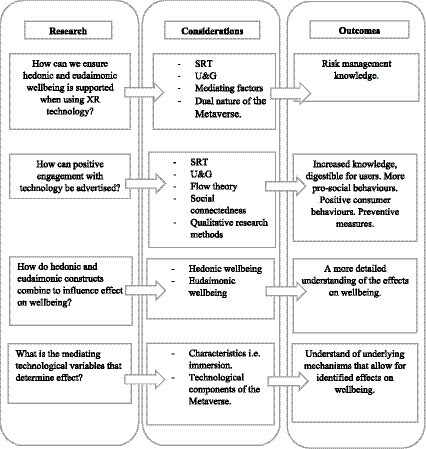
Future research agenda framework.

(1) How can we ensure hedonic and eudaimonic wellbeing is supported when using XR technology? For this, researchers should consider social, emotional, psychological, and cognitive contexts of wellbeing. So to describe use that ensures positive experiences that meet the needs of each context. Moreover, considering the dual effect on negative and positive incidences, i.e., XR’s ability to both foster and risk social connectedness, a risk assessment of effects is necessary. This will outline areas of improvement that can influence ongoing Metaverse development. Moreover, positive use can be promoted through translating SRT theory and U&Gs theory into practicality. With SRT in mind, developers should create experiences that reduce stress. Likewise, researchers should investigate how users can use technology with the sole purpose of differing needs.

(2) How can positive engagement with technology be advertised? As users will have a more active role in their experiences, compared to more traditional technology use, it is important that they themselves are aware of how they can engage positively with technology. From a marketing perspective, marketers should illustrate the nuances of SRT, U&G, and Flow theory, in an educative manner. Similarly, characteristics (6.3) hint at effects already noted in the literature. These can be used to further inform the user of risks. Giving them the knowledge, they need to take preventative action. This may be assisted by qualitative research methods, as these can provide more detailed knowledge pertaining to the dos and don’ts of the Metaverse.

(3) How do hedonic and eudaimonic constructs combine to influence effect on wellbeing? This paper highlights that hedonic and eudaimonic effects are not isolated from one another. An integrated theory of wellbeing that acknowledges and demonstrates the interplay between hedonia and eudaimonia should be considered within a Metaverse context. This will not only extend theory into a new domain but also provide insight into how XR technology manages to holistically affect wellbeing. Moreso, researchers should use this to explain the overlaps found between wellbeing contexts.

(4) The preliminary nature of this study means that evidence is limited. To rectify this, future research should further explore the top 5 most mentioned theories to understand fully the effects on wellbeing and how this resulted in the characteristics listed in section 6.1. An uncovering of mediating variables is needed to fully understand how theory becomes practice. For instance, what is it about XR technology that facilitates in U&G, or what is it about technology that reduces stress? Or promotes social connectedness and so on. As immersion has already been hinted to as a causal mechanism, this may be a good place to start. Figure 3 congregates the above research agendas within a framework.

## 5 Conclusion

Using an amalgamation of bibliometric and SLR methods, the current research was able to answer RQ 1-6. In doing so, the paper provides an overview of research investigating the effects of XR technology on consumer wellbeing which can be used to inform Metaverse stakeholders of the potential risks and benefits of their choices and better promote positive psychology. In addition, the review is suggestive of future research directions which can extend and develop theory.

This paper acts as an interim until more academic research has been made in relation to the Metaverse. However, the breakdown of technology means that observations and suggestions for future directions can easily be adapted to the metaverse as the Metaverse is founded upon this technology.

## Author contributions

AT: Data curation, Formal analysis, Investigation, Methodology, Validation, Visualization, Writing – original draft. MD: Conceptualization, Investigation, Methodology, Project administration, Supervision, Validation, Writing – review & editing. TJ: Conceptualization, Investigation, Methodology, Project administration, Supervision, Validation, Writing – review & editing. JC: Data curation, Investigation, Methodology, Writing – original draft. OK: Resources, Validation, Writing – review & editing.
