# Supplementary material for: XR and mental wellbeing: state of the art and future research directions for the Metaverse
Source: Front Psychol. 2024 Mar 8;15:1360260. doi: 10.3389/fpsyg.2024.1360260 (PMC10959091; doi:10.3389/fpsyg.2024.1360260)
Supplement: Supplementary file 1 [file Data_Sheet_1.docx]

**Appendices**

**Appendix 1 – Overview of bibliometric analysis**


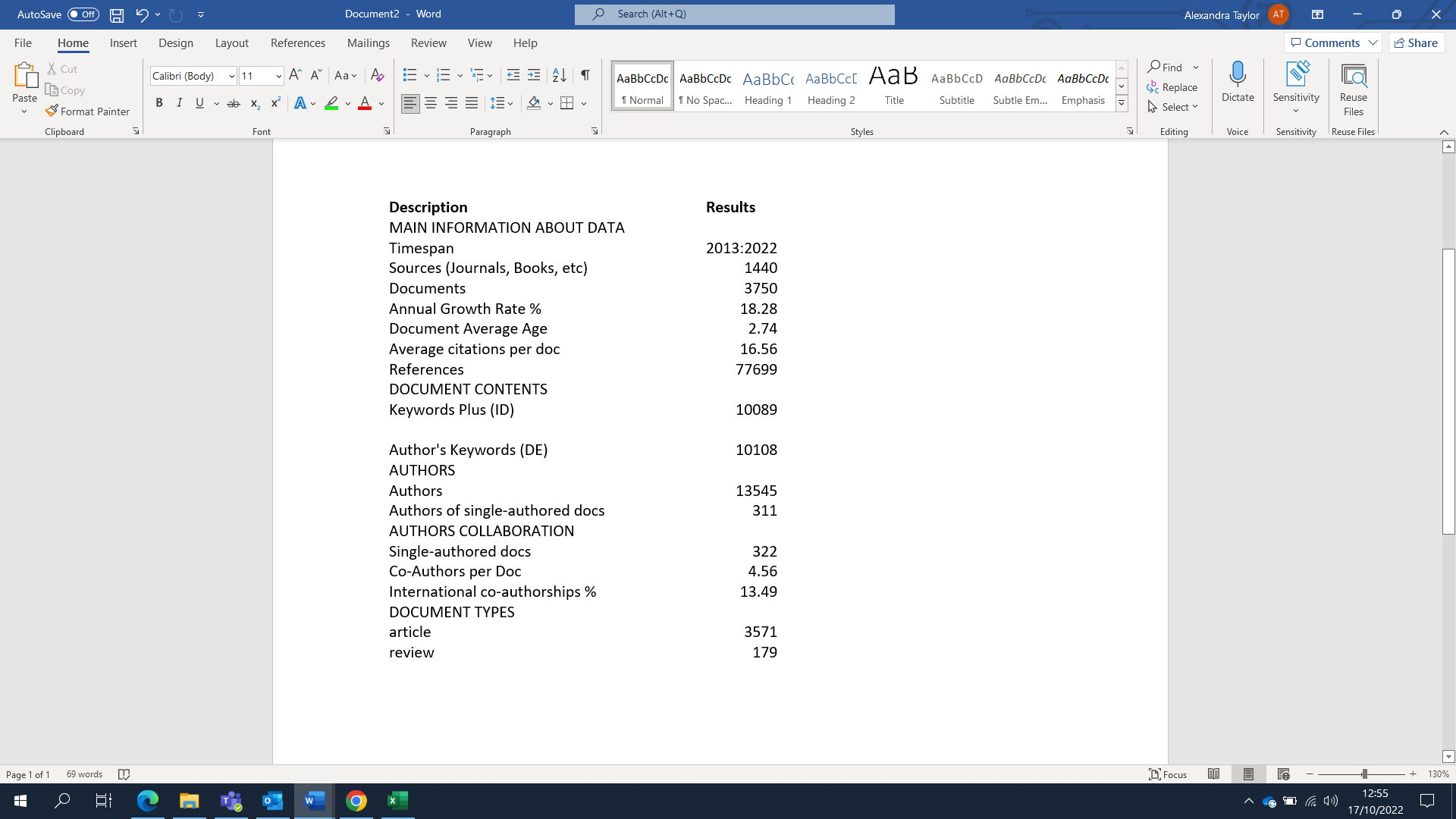


**Appendix 2 – Most impactful journal articles**


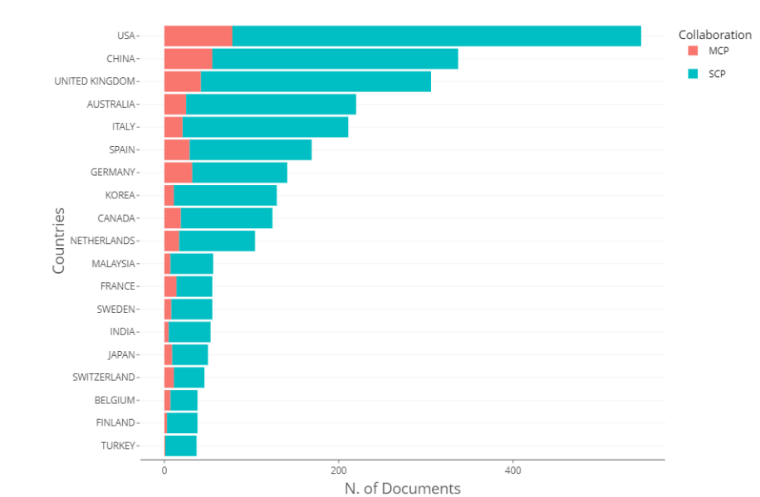


**Appendix 3– Scopus and Wos search strings conducted on 02/09/22**


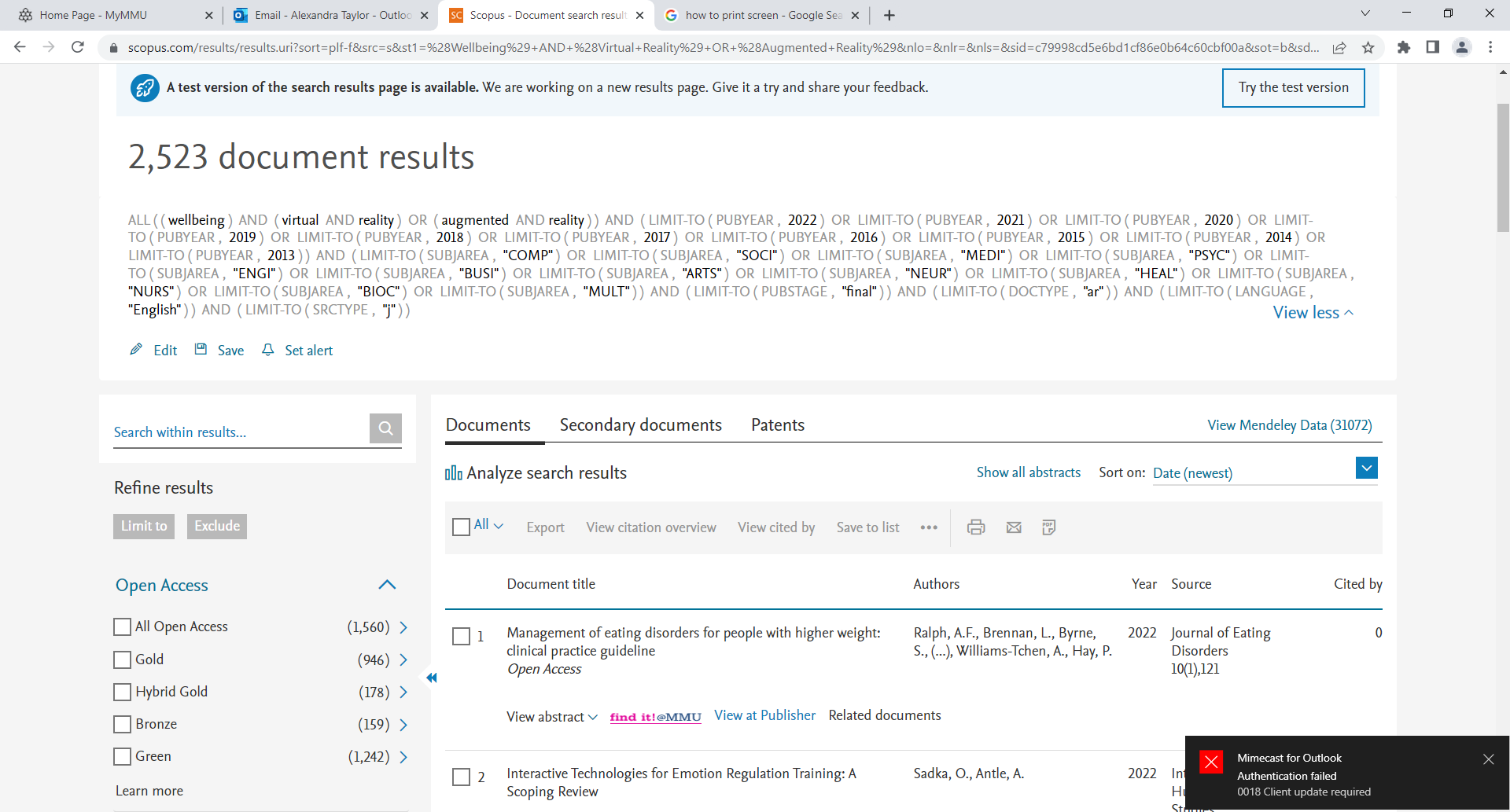


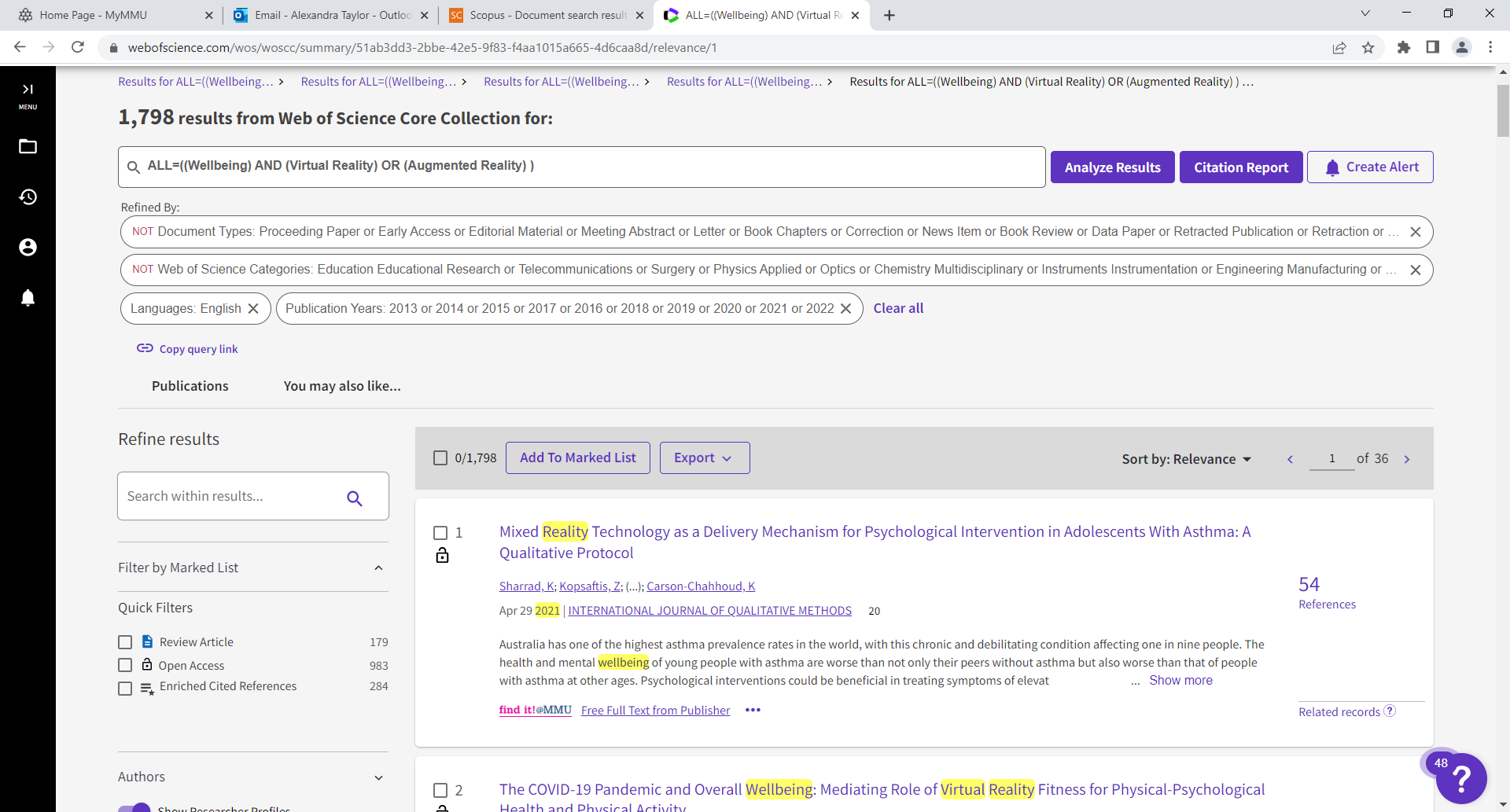


**Appendix 4 – initial inclusion/ exclusion criteria**

| Inclusion | Exclusion |
| --- | --- |
| Past 10 years (2013-2022) | Over 10 years |
| Journal articles | Other forms of publication |
| Final publication | Not final publication |
| English | Any other language |
| Topics relating to psychology, sociology, business, general health applications, psychiatry, biology, medical research, and computer science applications. | Irrelevant topics which do not relate to the research topic or questions |

**Appendix 5- PRISMA Methodology**

Duplicates Removed

Intra-databases (N=2)

Inter-databases (N= 66)

Potentially relevant articles reviewed in first screening

N= 4253

Articles excluded with reason based on title and key words

N= 3743

n

SCOPUS search.

(Wellbeing) AND (Virtual Reality) OR (Augmented Reality)

N= 2523

Web of Science search.

(Wellbeing) AND (Virtual Reality) OR (Augmented Reality)

N= 1798

Potentially relevant articles identified

N= 4321

Total studies meeting inclusion/ exclusion criteria

N= 81

Potentially relevant articles reviewed in second screening

N= 510

Articles excluded with reason based on abstract

N= 342

Or no abstract

N= 5

Potentially relevant articles reviewed third screening

N= 163

Articles excluded with reason based on restricted access (N= 7) or full text (N=75)

**Appendix 6– Synthesis of Methodology**

| Method | N |
| --- | --- |
| Quantitative | 46 |
| Qualitative | 22 |
| Mixed methods | 12 |
| Data mining | 1 |

**Appendix 7– Overview of theories**

| Topic area | Theory |
| --- | --- |
| Social psychological. | - Social Identity Theory - Social Influence - Socio-cognitive theory - Deindividuation - Psychosocial theory - Temporal need threat model - Social-trust theory - Social Self-efficacy theory - Compliance gaining theory - Media equation concept   Social categorisation theory |
| Emotional | - Mood induction process - Hedonic wellbeing. - Mindfulness - Buddhist psychology - Broaden and Build theory - Separation anxiety - PERMA - Positive psychology - Psycho-evolutionary theory |
| Functioning and behaviour. | - Eudaimonic wellbeing - Elaborated Intrusion theory - Socio-emotional behavioural functioning - Trans-theoretical model of behaviour change - Behavioural learning theory |
| Engagement and motivation. | - Flow - Self-determination theory - Dualistic model of passion - Technology acceptance model |
| Satisfaction | - Uses and Gratification theory - Social need gratification - Structured association technique - Humanistic needs theory - Leisure satisfaction - Utilitarian |
| Cognitive | - Mental Imagery theory - Psychoanalytical theory of unconsciousness - Theory of planned behaviour - Means end-chain theory - Misinformation effect - Cognitive theory - Cognitive emotion theory - Transient hypo frontality theory |
| Assistive technology | - Human-computer interactions - Theory of supportive design - Technology mediated therapeutic process - Computer supported collaborative learning |
| Self | - Temporarily expanding the boundaries of self - Ideal-actual gap - Bodily self-consciousness - Self-congruence theory - Augmented self - Perceived behavioural control - Computer-mediated self-transcendence - Self-affirmation theory - Transformative experiences |
| Restorative | - Attention restoration theory - Stress reduction theory - Biophilia - Salutogenesis - Theory of working alliance |
